# Crystal structure and Hirshfeld surface analysis of ethyl 5-phenyl­isoxazole-3-carboxyl­ate

**DOI:** 10.1107/S2056989017003127

**Published:** 2017-03-17

**Authors:** Althaf Shaik, Sivapriya Kirubakaran, Vijay Thiruvenkatam

**Affiliations:** a Department of Chemistry, IIT Gandhinagar, Gujarat; b Department of Physics & Bio-Engineering, IIT Gandhinagar, Palaj Campus, Gandhinagar, Gujarat

**Keywords:** crystal structure, isoxazole derivative, drug inter­mediate, Hirshfeld surface, hydrogen bonding

## Abstract

The title compound, C_12_H_11_NO_3_, is an inter­mediate used in the synthesis of many drug-like mol­ecules. The mol­ecule is almost planar, with the phenyl ring inclined to the isoxazole ring by 0.5 (1)°. The ester moiety has an extended conformation and is almost in the same plane with respect to the isoxazole ring, as indicated by the O—C—C—N torsion angle of −172.86 (18)°. In the crystal, mol­ecules are linked *via* pairs of C—H⋯O hydrogen bonds with the same acceptor atom, forming inversion dimers with two *R*_2_^1^(7) ring motifs. The mol­ecules stack in layers lying parallel to (10-3). Analysis using Hirshfeld surface generation and two-dimensional fingerprint plots explores the distribution of weak inter­molecular inter­actions in the crystal structure.

## Abstract 2

In the title isoxazole derivative, the phenyl and isoxazole rings are in the same plane, as indicated by the C—C—C—O torsion angle of 0.1 (3)°. The ester group has an extended conformation and is almost in the same plane with respect to the isoxazole ring, as indicated by the O—C—C—N torsion angle of −172.86 (18)°.

## Chemical context   

Nitro­gen-containing heterocyclic rings are of great importance in medicinal and organic chemistry (Dou *et al.*, 2013 ▸). Isoxazole derivatives are important heterocyclic pharmaceuticals having a broad spectrum of biological activity, which includes antagonism of the NMDA receptor, anti-inflammatory (Panda *et al.*, 2009 ▸), anti-tumour, anti­convulsant, anti-psychotic, anti-depressant and anti HIV activity (Conti *et al.*, 2005 ▸; Srivastava *et al.*, 1999 ▸). Considerable attention has been paid to isoxazole derivatives as a result of their prominent biological properties (Dou *et al.*, 2013 ▸). Valdecoxib (Bextra), a selective cyclo­oxygenase-2 (COX-2) inhibitor used in the treatment of arthritis, contains an isoxazole moiety which is responsible for its biological activity (Waldo & Larock, 2007 ▸; Dadiboyena & Nefzi, 2010 ▸). In addition, isoxazole derivatives are also important inter­mediates in the preparation of various heterocyclic biologically active drugs (Dou *et al.*, 2013 ▸). As part of our ongoing studies on isoxazole derivatives as kinase inhib­itors, we have synthesized the title compound, and report herein on its crystal structure and the qu­anti­tative analysis of inter­molecular inter­actions using the Hirshfeld surface and 2D fingerprint plot analysis.
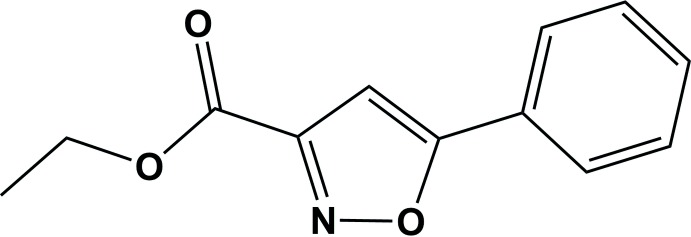


## Structural commentary   

**Figure 1:**
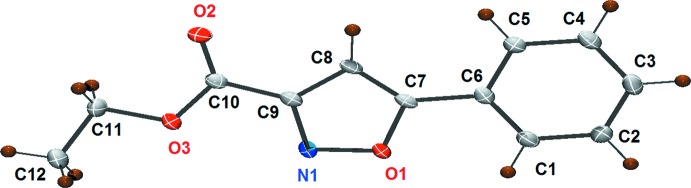
The mol­ecular structure of compound (I), with the atom labelling and displacement ellipsoid drawn at the 50% probability level.

The mol­ecular structure of the title compound, (I), is illus­trated in Fig. 1 ▸. The mol­ecule consists of three almost flat units: the phenyl ring, the isoxazole ring and the ester. The phenyl (C1–C6) and isoxazole (O1/N1/C7–C9) rings are almost coplanar, as indicated by the torsion angle C5—C6—C7—O1 = 0.1 (3)°. The ester unit has an extended conformation and is almost in the same plane as the isoxazole ring, as indicated by the torsion angle O2—C10—C9—N1 = −172.86 (18)°.

## Supra­molecular features   

**Figure 2:**
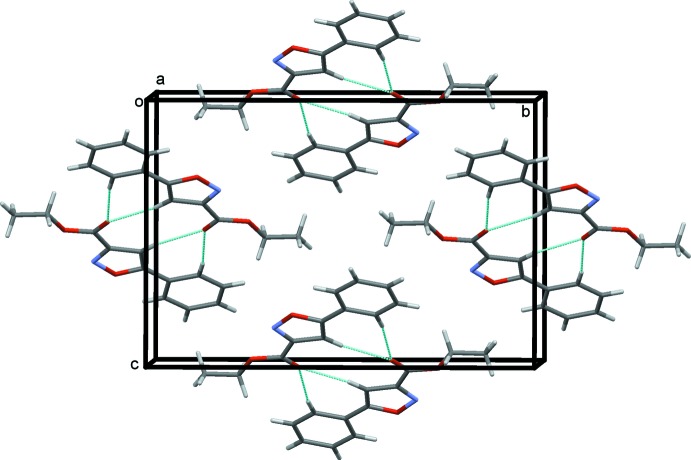
Crystal packing of compound (I), viewed along the *a* axis. Hydrogen bonds are shown as dashed lines (see Table 1 ▸).

**Figure 3:**
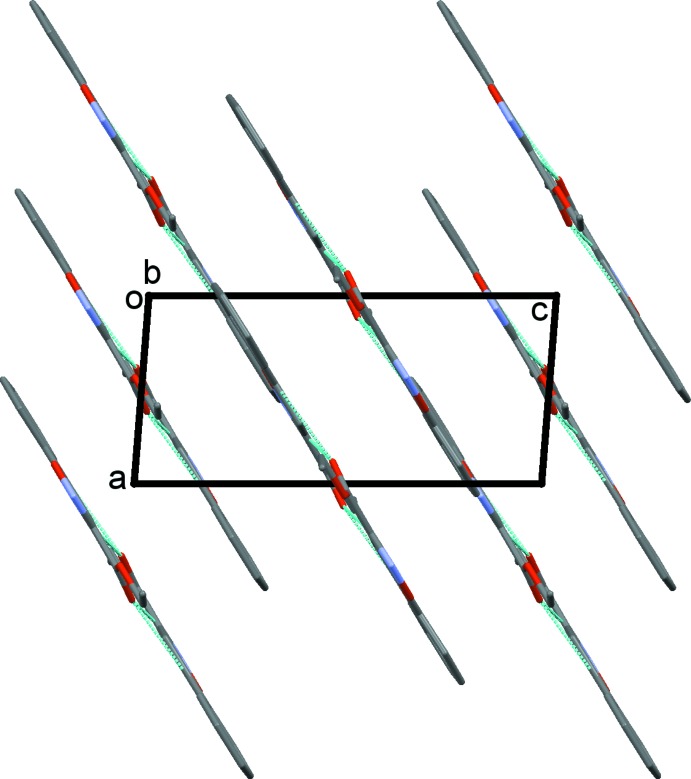
Crystal packing of compound (I) viewed along the *b* axis. Hydrogen bonds are shown as dashed lines and, for clarity, H atoms have been omitted.

In the crystal of (I), mol­ecules are linked *via* pairs of C—H⋯O hydrogen bonds, both involving atom O2 as acceptor, forming inversion dimers with two 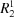
(7) ring motifs (Table 1 ▸ and Fig. 2 ▸). The mol­ecules stack in layers lying parallel to (10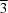), as illustrated in Fig. 3 ▸.

## Hirshfeld surface and fingerprint plot analysis   

**Figure 4:**
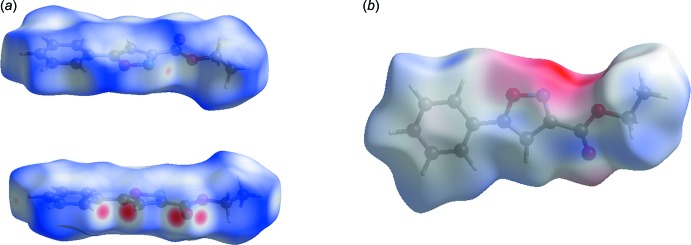
Hirshfeld surface mapped over (*a*) *d*_norm_ and (*b*) electrostatic potential.

**Figure 5:**
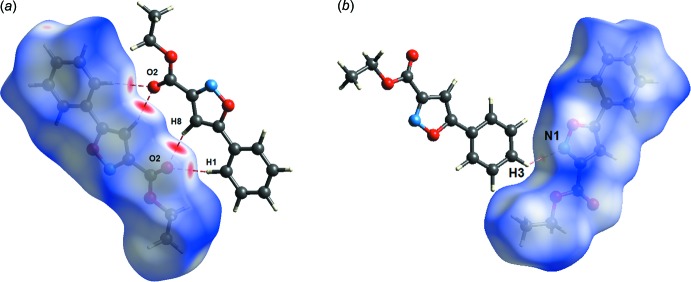
Hirshfeld surface mapped over (*a*) *d*_norm_ highlighting the regions of C—H⋯O hydrogen bonding and (*b*) *d*_norm_ highlighting the region of C—H⋯N hydrogen bonding.

**Figure 6:**
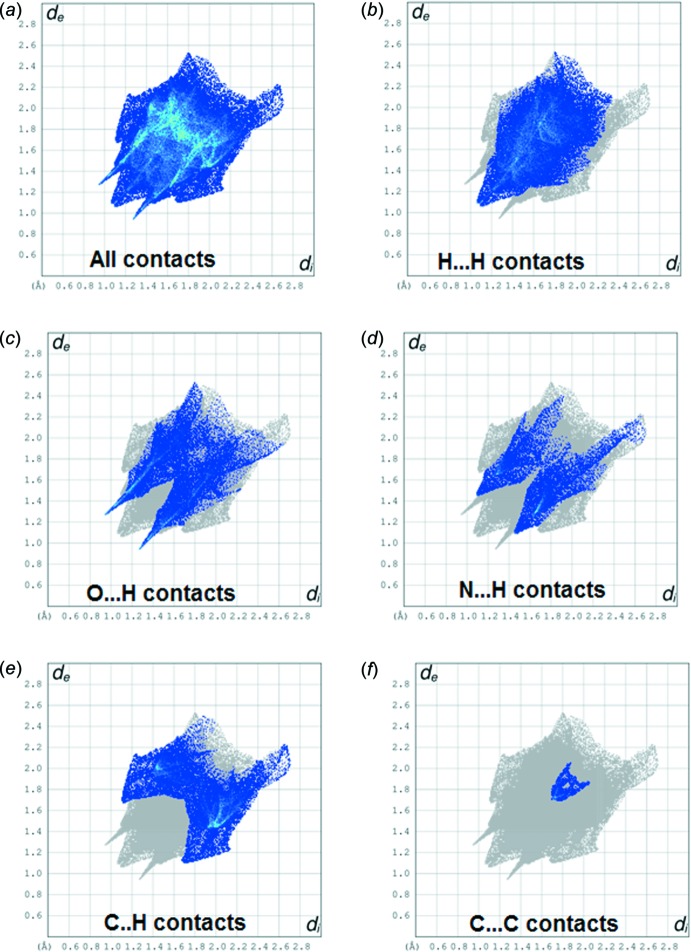
Two-dimensional fingerprint plot analysis (*a*) all inter­actions, (*b*) H⋯H contacts, (*c*) O⋯H contacts, (*d*) N⋯H contacts, (*e*) C⋯H contacts and (*f*) C⋯C contacts.

To explore the weak inter­molecular inter­actions in (I), Hirshfeld surfaces and 2D fingerprint plots were generated using *Crystal Explorer 3.1* to qu­antify the inter­molecular inter­actions (McKinnon *et al.*, 2007 ▸; Spackman & Jayatilaka, 2009 ▸). Hirshfeld surfaces are produced through the partitioning of space within a crystal where the ratio of promol­ecule to procrystal electron density is equal to 0.5, generating continuous, non-overlapping surfaces which are widely used to visualize and study the significance of weak inter­actions in the mol­ecular packing (McKinnon *et al.*, 2007 ▸). The Hirshfeld surface of title compound was mapped over *d*_norm_, shape index and curvedness. The *d*_norm_ surface is the normalized function of *d*_i_ and *d*_e_ (Fig. 4 ▸ *a*), with white-, red- and blue-coloured surfaces. The white surface indicates those contacts with distances equal to the sum of the van der Waals (vdW) radii, red indicates shorter contacts (< vdW radii) and blue the longer contact (> vdW radii). The Hirshfeld surface was also mapped over electrostatic potential (Fig. 4 ▸ *b*) using a STO-3G basis set at the Hartee–Fock level of theory (Spackman & McKinnon, 2002 ▸; McKinnon *et al.*, 2004 ▸). In the Hirshfeld surface, a pair of inter­actions between the aromatic C—H⋯O=C atoms can be seen as the bright-red area (1) in Fig. 5 ▸ *a*. The 2D fingerprint plot analysis of the O⋯H inter­actions revealed significant hydrogen-bonding spikes (*d*_i_ = 1.3, *d*_e_ = 0.9 Å and *d*_e_ = 1.9, *d*_i_ = 2.6 Å); Fig. 6 ▸ *c*.

The analysis indicates that there is a weak N⋯H inter­molecular inter­action between the nitro­gen atom of the isoxazole ring and the methyl­ene hydrogen atom of the phenyl ring of a neighbouring mol­ecule (Fig. 5 ▸ *b*). The fingerprint plot analysis of N⋯H contacts reveals a significant wing-like structure (*d*_i_ = 1.2, *d*_e_ = 1.5 Å and *d*_e_ = 2.2, *d*_i_ = 2.4 Å) Fig. 6 ▸ *d*.

The relative contributions to the Hirshfeld surface area for each type of inter­molecular contact are illustrated in Figs. 6 ▸ and 7 ▸. The H⋯H inter­actions appear as scattered points over nearly the entire plot and have a significant composition of 41% of the Hirshfeld surface. The H⋯O contacts comprise of 18.7% and the C⋯C inter­actions comprise 1.6% of the total Hirshfeld surface. The C⋯H and N⋯H inter­actions cover 23.2% and 9.2% of the surface, respectively. Thus, these weak inter­actions contribute significantly to the packing of (I).

## Database survey   

A search of the Cambridge Structural Database (CSD, V5.38; last update November 2016; Groom *et al.*, 2016 ▸) for similar isoxazole derivatives, revealed only one hit, *viz*. ethyl 5-(4-amino­phen­yl) isoxazole-3-carboxyl­ate (CSD refcode YAVRIY; Zhao *et al.*, 2012 ▸). This compound crystallizes with two independent mol­ecules in the asymmetric unit. One mol­ecule is slightly more planar than the other, with the phenyl ring being inclined to the isoxazole ring by 1.77 (10) and 5.85 (10)°. In the title compound, (I), this dihedral angle is 0.5 (1)°.

## Synthesis and crystallization   

**Figure 8:**
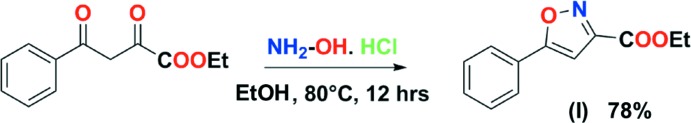
Synthesis of the title compound, (I).

There are several methods available in the literature for the preparation of isoxazole derivatives. We have followed a simple preparation from a diketoester (Tourteau *et al.*, 2013 ▸; Bastos *et al.*, 2015 ▸). After the reaction of aceto­phenone with diethyoxalate in a basic solution (sodium ethoxide) of ethanol for 8 h, 1*N* HCl was added to neutralize the sodium ethoxide to obtain the diketoester (ethyl 2,4-dioxo-4-phenyl­butano­ate; see Fig. 8 ▸) as a yellow liquid. 1 g (4.5 mmol) of the diketoester in ethanol was added to hydroxyl amine hydro­chloride (0.315 g, 4.5 mmol) at room temperature and the resulting mixture was stirred at 353 K for 12 h. The progress of the reaction was monitored by TLC. After the completion of starting materials, the reaction mixture was cooled to room temperature and the excess of ethanol removed. The resulting residue was dissolved in water and extracted with ethyl acetate. The organic layer was dried with Na_2_SO_4_, filtered and the concentrated under reduced pressure. The resulting residue was purified by silica gel column chromatography (3% ethyl acetate: Pet-ether) to afford the title compound, (I) (yield 76.9%, 0.75 g; m.p. 325–327 K).

Colourless crystals were obtained by slow evaporation of a solution in ethyl acetate. Spectroscopic data: ^1^H NMR (500 MHz, chloro­form-*d*) δ 7.80 (*m*, 2H), 7.50 (*m*, 3H), 6.92 (*s*, 1H), 4.47 (*q*, 2H), 1.44 (*t*, 3H). ^13^C NMR (126 MHz, chloro­form-*d*) δ 171.66, 159.98, 156.96, 130.76, 129.11, 126.61, 125.89, 99.92, 62.18, 14.15.

## Refinement   

Crystal data, data collection and structure refinement parameters are given in Table 2 ▸. All H atoms were positioned geometrically and refined as riding: C—H = 0.95–0.99 Å with *U*_iso_(H) = 1.2*U*_eq_(C).

## Supplementary Material

Crystal structure: contains datablock(s) I, Global. DOI: 10.1107/S2056989017003127/su5350sup1.cif

Structure factors: contains datablock(s) I. DOI: 10.1107/S2056989017003127/su5350Isup2.hkl

Supporting information file. DOI: 10.1107/S2056989017003127/su5350Isup5.cml

Supporting information file. DOI: 10.1107/S2056989017003127/su5350sup3.png

Supporting information file. DOI: 10.1107/S2056989017003127/su5350sup4.png

CCDC reference: 1534636

Additional supporting information:  crystallographic information; 3D view; checkCIF report

## Figures and Tables

**Figure 7:**
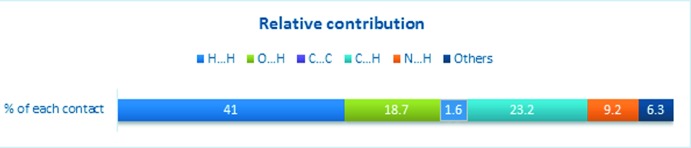
Relative contribution of each inter­action in the two-dimensional fingerprint analysis.

**Table 1:** Hydrogen-bond geometry (Å, °)

*D*—H⋯*A*	*D*—H	H⋯*A*	*D*⋯*A*	*D*—H⋯*A*
C1—H1⋯O2^i^	0.93	2.52	3.447 (2)	171
C8—H8⋯O2^i^	0.93	2.36	3.260 (2)	163

Symmetry code: (i) 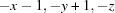.

**Table 2:** Experimental details

Crystal data	
Chemical formula	C_12_H_11_NO_3_
*M*_r_	217.22
Crystal system, space group	Monoclinic, *P*2_1_/*n*
Temperature (K)	100
*a*, *b*, *c* (Å)	5.4447 (7), 17.180 (2), 11.7603 (19)
β (°)	94.508 (5)
*V* (Å^3^)	1096.6 (3)
*Z*	4
Radiation type	Mo *K*α
μ (mm^−1^)	0.10
Crystal size (mm)	0.4 × 0.2 × 0.2

Data collection	
Diffractometer	Bruker APEXII CCD
Absorption correction	–
No. of measured, independent and observed [*I* > 2σ(*I*)] reflections	14119, 2813, 1889
*R*_int_	0.075
(sin θ/λ)_max_ (Å^−1^)	0.676

Refinement	
*R*[*F* ^2^ > 2σ(*F* ^2^)], *wR*(*F* ^2^), *S*	0.064, 0.177, 1.09
No. of reflections	2813
No. of parameters	146
H-atom treatment	H-atom parameters constrained
Δρ_max_, Δρ_min_ (e Å^−3^)	0.27, −0.30

Computer programs: *APEX2* and *SAINT* (Bruker, 2006 ▸), *SHELXS97* and *SHELXTL* (Sheldrick 2008 ▸), *SHELXL2014* (Sheldrick, 2015 ▸), and *Mercury* (Macrae *et al.*, 2008 ▸).

## supplementary crystallographic information

### Crystal data

**Table:**

C_12_H_11_NO_3_	*F*(000) = 456
*M**_r_* = 217.22	*D*_x_ = 1.316 Mg m^−^^3^
Monoclinic, *P*2_1_/*n*	Mo *K*α radiation, λ = 0.71073 Å
*a* = 5.4447 (7) Å	Cell parameters from 5392 reflections
*b* = 17.180 (2) Å	θ = 2.4–30.5°
*c* = 11.7603 (19) Å	µ = 0.10 mm^−^^1^
β = 94.508 (5)°	*T* = 100 K
*V* = 1096.6 (3) Å^3^	Blocks, colourless
*Z* = 4	0.4 × 0.2 × 0.2 mm

### Data collection

**Table:**

Bruker APEXII CCD diffractometer	*R*_int_ = 0.075
φ and ω scans	θ_max_ = 28.7°, θ_min_ = 2.4°
14119 measured reflections	*h* = −5→7
2813 independent reflections	*k* = −23→23
1889 reflections with *I* > 2σ(*I*)	*l* = −15→14

### Refinement

**Table:**

Refinement on *F*^2^	Primary atom site location: structure-invariant direct methods
Least-squares matrix: full	Secondary atom site location: difference Fourier map
*R*[*F*^2^ > 2σ(*F*^2^)] = 0.064	Hydrogen site location: inferred from neighbouring sites
*wR*(*F*^2^) = 0.177	H-atom parameters constrained
*S* = 1.09	*w* = 1/[σ^2^(*F*_o_^2^) + (0.1*P*)^2^] where *P* = (*F*_o_^2^ + 2*F*_c_^2^)/3
2813 reflections	(Δ/σ)_max_ < 0.001
146 parameters	Δρ_max_ = 0.27 e Å^−^^3^
0 restraints	Δρ_min_ = −0.30 e Å^−^^3^

### Special details

**Table:**

Geometry. All esds (except the esd in the dihedral angle between two l.s. planes) are estimated using the full covariance matrix. The cell esds are taken into account individually in the estimation of esds in distances, angles and torsion angles; correlations between esds in cell parameters are only used when they are defined by crystal symmetry. An approximate (isotropic) treatment of cell esds is used for estimating esds involving l.s. planes.

### Fractional atomic coordinates and isotropic or equivalent isotropic displacement parameters (Å^2^)

**Table:**

	*x*	*y*	*z*	*U*_iso_*/*U*_eq_	
O1	0.1048 (2)	0.62026 (7)	0.20002 (11)	0.0221 (4)	
O3	−0.4360 (3)	0.73867 (7)	0.01862 (12)	0.0230 (4)	
O2	−0.6161 (2)	0.62208 (7)	−0.01700 (11)	0.0243 (4)	
C10	−0.4499 (3)	0.66119 (10)	0.02530 (16)	0.0184 (4)	
C6	0.1914 (3)	0.48387 (10)	0.22525 (16)	0.0181 (4)	
N1	−0.0666 (3)	0.67215 (8)	0.14685 (14)	0.0225 (4)	
C4	0.5520 (4)	0.44302 (11)	0.34137 (19)	0.0256 (5)	
H4	0.692513	0.455560	0.387897	0.031*	
C7	0.0322 (4)	0.54579 (9)	0.17410 (16)	0.0174 (4)	
C9	−0.2320 (3)	0.62718 (10)	0.09196 (15)	0.0180 (4)	
C5	0.4000 (4)	0.50161 (10)	0.29503 (17)	0.0234 (5)	
H5	0.438869	0.553372	0.311094	0.028*	
C3	0.4941 (4)	0.36559 (11)	0.31817 (17)	0.0249 (5)	
H3	0.596502	0.326217	0.348598	0.030*	
C1	0.1308 (4)	0.40540 (10)	0.20262 (16)	0.0203 (4)	
H1	−0.010182	0.392615	0.156628	0.024*	
C12	−0.5855 (4)	0.86283 (11)	−0.04174 (18)	0.0273 (5)	
H12A	−0.579681	0.880375	0.035929	0.041*	
H12B	−0.711938	0.890614	−0.086440	0.041*	
H12C	−0.429204	0.872225	−0.071751	0.041*	
C8	−0.1780 (3)	0.54715 (9)	0.10639 (16)	0.0185 (4)	
H8	−0.267753	0.504983	0.075862	0.022*	
C11	−0.6410 (4)	0.77721 (11)	−0.04635 (18)	0.0243 (5)	
H11A	−0.794720	0.766468	−0.012878	0.029*	
H11B	−0.654755	0.759053	−0.124690	0.029*	
C2	0.2846 (4)	0.34724 (10)	0.24996 (17)	0.0237 (5)	
H2	0.245566	0.295307	0.235394	0.028*	

### Atomic displacement parameters (Å^2^)

**Table:**

	*U*^11^	*U*^22^	*U*^33^	*U*^12^	*U*^13^	*U*^23^
O1	0.0236 (8)	0.0114 (6)	0.0300 (8)	0.0009 (5)	−0.0065 (6)	0.0016 (5)
O3	0.0239 (8)	0.0128 (6)	0.0314 (8)	0.0024 (5)	−0.0047 (6)	0.0009 (5)
O2	0.0233 (8)	0.0170 (6)	0.0319 (8)	−0.0022 (5)	−0.0028 (6)	−0.0018 (6)
C10	0.0199 (10)	0.0140 (8)	0.0215 (10)	−0.0001 (7)	0.0026 (8)	−0.0025 (7)
C6	0.0190 (10)	0.0150 (8)	0.0207 (10)	0.0016 (7)	0.0047 (8)	0.0019 (7)
N1	0.0246 (10)	0.0139 (7)	0.0280 (9)	0.0031 (6)	−0.0052 (7)	0.0029 (7)
C4	0.0195 (11)	0.0220 (10)	0.0345 (12)	−0.0005 (8)	−0.0036 (8)	0.0034 (8)
C7	0.0234 (11)	0.0102 (8)	0.0190 (10)	−0.0019 (7)	0.0041 (8)	−0.0019 (7)
C9	0.0209 (10)	0.0131 (8)	0.0204 (10)	−0.0019 (7)	0.0035 (8)	0.0001 (7)
C5	0.0211 (11)	0.0146 (9)	0.0345 (12)	−0.0003 (7)	0.0017 (9)	0.0006 (8)
C3	0.0224 (11)	0.0195 (9)	0.0331 (11)	0.0057 (8)	0.0038 (9)	0.0064 (8)
C1	0.0218 (11)	0.0161 (8)	0.0228 (10)	−0.0003 (7)	0.0005 (8)	−0.0010 (7)
C12	0.0294 (12)	0.0197 (9)	0.0320 (12)	0.0047 (8)	−0.0019 (9)	0.0042 (8)
C8	0.0208 (10)	0.0108 (8)	0.0243 (10)	−0.0014 (7)	0.0032 (8)	−0.0020 (7)
C11	0.0219 (11)	0.0197 (9)	0.0300 (11)	0.0041 (8)	−0.0055 (8)	0.0009 (8)
C2	0.0276 (11)	0.0150 (8)	0.0289 (11)	0.0020 (8)	0.0042 (8)	−0.0001 (8)

### Geometric parameters (Å, º)

**Table:**

O1—C7	1.366 (2)	C9—C8	1.413 (2)
O1—N1	1.4018 (19)	C5—H5	0.9300
O3—C10	1.336 (2)	C3—C2	1.379 (3)
O3—C11	1.461 (2)	C3—H3	0.9300
O2—C10	1.203 (2)	C1—C2	1.391 (3)
C10—C9	1.489 (3)	C1—H1	0.9300
C6—C5	1.382 (3)	C12—C11	1.502 (2)
C6—C1	1.408 (2)	C12—H12A	0.9600
C6—C7	1.471 (2)	C12—H12B	0.9600
N1—C9	1.317 (2)	C12—H12C	0.9600
C4—C3	1.389 (3)	C8—H8	0.9300
C4—C5	1.387 (3)	C11—H11A	0.9700
C4—H4	0.9300	C11—H11B	0.9700
C7—C8	1.342 (3)	C2—H2	0.9300
			
C7—O1—N1	108.98 (13)	C4—C3—H3	120.1
C10—O3—C11	115.97 (14)	C2—C1—C6	119.14 (18)
O2—C10—O3	125.19 (16)	C2—C1—H1	120.4
O2—C10—C9	122.75 (16)	C6—C1—H1	120.4
O3—C10—C9	112.06 (15)	C11—C12—H12A	109.5
C5—C6—C1	119.53 (17)	C11—C12—H12B	109.5
C5—C6—C7	120.93 (16)	H12A—C12—H12B	109.5
C1—C6—C7	119.54 (17)	C11—C12—H12C	109.5
C9—N1—O1	104.55 (14)	H12A—C12—H12C	109.5
C3—C4—C5	119.89 (19)	H12B—C12—H12C	109.5
C3—C4—H4	120.1	C9—C8—C7	104.35 (15)
C5—C4—H4	120.1	C9—C8—H8	127.8
O1—C7—C8	109.53 (15)	C7—C8—H8	127.8
O1—C7—C6	115.78 (16)	O3—C11—C12	106.35 (15)
C8—C7—C6	134.68 (16)	O3—C11—H11A	110.5
C8—C9—N1	112.59 (16)	C12—C11—H11A	110.5
C8—C9—C10	126.49 (16)	O3—C11—H11B	110.5
N1—C9—C10	120.92 (15)	C12—C11—H11B	110.5
C6—C5—C4	120.69 (17)	H11A—C11—H11B	108.7
C6—C5—H5	119.7	C3—C2—C1	120.87 (17)
C4—C5—H5	119.7	C3—C2—H2	119.6
C2—C3—C4	119.87 (18)	C1—C2—H2	119.6
C2—C3—H3	120.1		
			
C11—O3—C10—O2	−0.4 (3)	O3—C10—C9—N1	7.3 (2)
C11—O3—C10—C9	179.40 (15)	C1—C6—C5—C4	1.1 (3)
C7—O1—N1—C9	−0.10 (19)	C7—C6—C5—C4	−178.82 (18)
N1—O1—C7—C8	0.15 (19)	C3—C4—C5—C6	−0.4 (3)
N1—O1—C7—C6	−179.04 (15)	C5—C4—C3—C2	−0.6 (3)
C5—C6—C7—O1	0.1 (3)	C5—C6—C1—C2	−0.9 (3)
C1—C6—C7—O1	−179.73 (15)	C7—C6—C1—C2	179.02 (17)
C5—C6—C7—C8	−178.8 (2)	N1—C9—C8—C7	0.1 (2)
C1—C6—C7—C8	1.3 (3)	C10—C9—C8—C7	−178.89 (17)
O1—N1—C9—C8	0.0 (2)	O1—C7—C8—C9	−0.13 (19)
O1—N1—C9—C10	179.04 (15)	C6—C7—C8—C9	178.9 (2)
O2—C10—C9—C8	6.0 (3)	C10—O3—C11—C12	179.89 (15)
O3—C10—C9—C8	−173.78 (16)	C4—C3—C2—C1	0.8 (3)
O2—C10—C9—N1	−172.86 (18)	C6—C1—C2—C3	0.0 (3)

### Hydrogen-bond geometry (Å, º)

**Table:**

*D*—H···*A*	*D*—H	H···*A*	*D*···*A*	*D*—H···*A*
C1—H1···O2^i^	0.93	2.52	3.447 (2)	171
C8—H8···O2^i^	0.93	2.36	3.260 (2)	163

Symmetry code: (i) −*x*−1, −*y*+1, −*z*.
