# Supplementary figures and images for: Crystal structure and Hirshfeld surface analysis of ethyl 5-phenyl­isoxazole-3-carboxyl­ate
Source: Acta Crystallogr E Crystallogr Commun. 2017 Mar 17;73(Pt 4):531–4. doi: 10.1107/S2056989017003127 (PMC5382615; doi:10.1107/S2056989017003127)

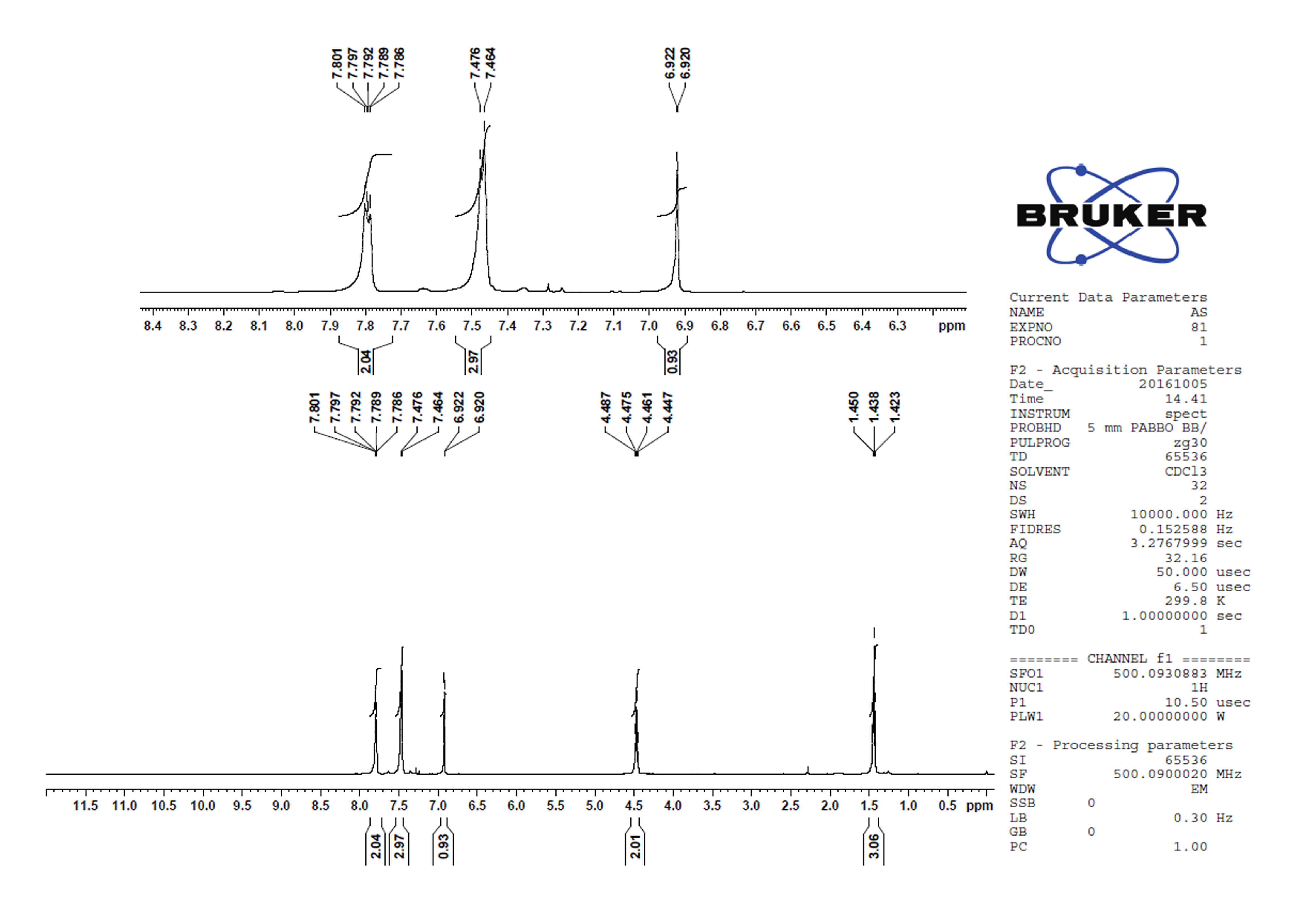

Supplement: Supplementary file 4 [file e-73-00531-sup3.png]

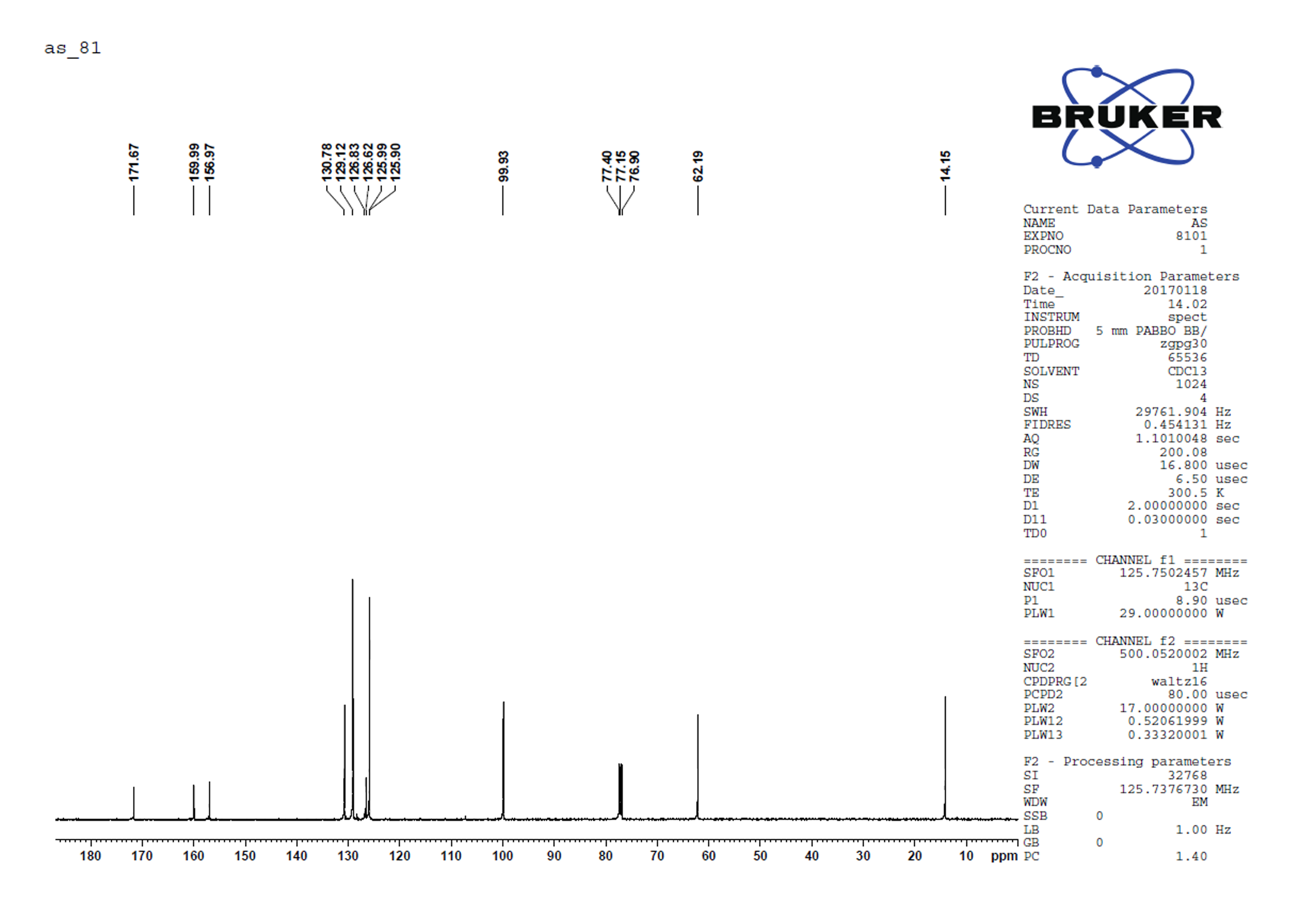

Supplement: Supplementary file 5 [file e-73-00531-sup4.png]
